# The Complexities and Nuances of Analyzing the Genome of *Drosophila ananassae* and Its *Wolbachia* Endosymbiont

**DOI:** 10.1534/g3.117.300164

**Published:** 2018-01-04

**Authors:** Julie C. Dunning Hotopp, Lisa Klasson

**Affiliations:** * Institute for Genome Sciences, Department of Microbiology and Immunology, Greenebaum Cancer Center, University of Maryland School of Medicine, Baltimore, Maryland 21201; † Molecular Evolution, Department of Cell and Molecular Biology, Uppsala University, 75124 Sweden

## Abstract

In “Retrotransposons Are the Major Contributors to the Expansion of the *Drosophila ananassae* Muller F Element,” Leung *et al.* (2017) improved contigs attributed to the Muller F element from the original CAF1 assembly, and used them to conclude that most of the sequence expansion of the fourth chromosome of *D. ananassae* is due to a higher transposon load than previously thought, but is not due to *Wolbachia* DNA integrations. While we do not disagree with the first conclusion, the authors base their second conclusion on the lack of homology detected between their improved CAF1 genome assembly attributed to *D. ananassae* and reference *Wolbachia* genomes. While the consensus CAF1 genome assembly lacks any sequence similarity to the reference genome of the *Wolbachia* endosymbiont of *Drosophila melanogaster* (*w*Mel), numerous studies from multiple laboratories provide experimental support for a large lateral/horizontal gene transfer (LGT) of a *Wolbachia* genome into this *D. ananassae* line. As such, we strongly suspect that the original whole genome assembly was either constructed after the removal of all *Wolbachia* reads, or that *Wolbachia* sequences were directly removed from the contigs in the CAF1 assembly. Hence, Leung *et al.* (2017) could not have identified the *Wolbachia* LGT using the CAF1 assembly. This manuscript by Leung *et al.* (2017) highlights that an assembly of the *Wolbachia* sequence reads and their mate pairs was erroneously attributed solely to the *Wolbachia* endosymbiont, albeit before we understood the extent of LGT in *D. ananassae*. As such, we recommend that the sequences deposited at the National Center for Biotechnology Information (NCBI) under PRJNA13365 should not be attributed to *Wolbachia* endosymbiont of *D. ananassae*, but should have their taxonomy reclassified by NCBI as “Unclassified sequences.” As our knowledge about genome biology improves, we need to reconsider and reanalyze earlier genomes removing the prejudice introduced from now defunct paradigms.

We were interested to read the recent paper by Leung *et al.* (2017) entitled “Retrotransposons Are the Major Contributors to the Expansion of the *Drosophila ananassae* Muller F Element.” Leung *et al.* (2017) use contigs attributed to the Muller F element from the original CAF1 assembly (Zimin *et al.* 2008), as well as improvements they made, to conclude that most of the sequence expansion of the fourth chromosome of *D. ananassae* is due to a higher transposon load than previously thought, but is not due to *Wolbachia* DNA integrations. Although we do not disagree with the first conclusion, we were surprised to see that the authors stated that the *Wolbachia* sequences integrated into the *D. ananassae* genome are a minor contributor to expansion of the Muller F Element. The authors base their conclusions on the lack of homology detected between their improvements to the CAF1 genome assembly attributed to *D. ananassae* and reference *Wolbachia* genomes. However, the CAF1 assembly was undertaken at a time when the dogma was that animal genomes did not contain lateral/horizontal gene transfer (LGT) from bacteria. As such, we strongly suspect that the original whole genome assembly was either constructed after the removal of all of the reads matching the closed/complete genome of the *Wolbachia* endosymbiont of *Drosophila melanogaster* (*w*Mel), the only *Wolbachia* genome available at the time, or that contigs in the assembly matching the closed/complete genome of the *Wolbachia* endosymbiont of *D. melanogaster* (*w*Mel) were removed from the two assemblies used to construct the CAF1 assembly. Despite our best efforts to clarify this by contacting as many of the assembly experts involved at that time as we could find, we cannot say definitively. However, this deduction is supported by the copious numbers of raw sequence reads with homology to *Wolbachia*, and that the only portions of *Wolbachia* sequence in the CAF1 assembly are those regions that do not share homology with the *w*Mel genome, as discussed previously (Klasson *et al.* 2009). Given the dogma at that time, it is reasonable that either of these approaches were undertaken, but unfortunately went unreported. Hence, Leung *et al.* (2017) could not have identified the *Wolbachia* LGT on the fourth or any chromosome of *D. ananassae* using the CAF1 assembly, as most of the *Wolbachia* sequences have been removed.

Furthermore, given that the original whole genome sequencing project on *D. ananassae* (Drosophila 12 Genomes Consortium *et al.* 2007) did not rely on genomic DNA prepared from embryos of an antibiotic-treated line to remove the *Wolbachia* endosymbionts (T. Markow, personal communication), we now understand that the “*Wolbachia*” sequence reads are a mixture of *Drosophila* and *Wolbachia* sequences. Unfortunately, given the very high similarity between the LGT and the residing bacterium, it is not possible to assign the reads to the *Drosophila* genome or the *Wolbachia* genome. This collective work on *D. ananassae* genomics, including this manuscript by Leung *et al.* (2017), highlights that an assembly of these sequences was erroneously attributed solely to the *Wolbachia* endosymbiont (Salzberg *et al.* 2005), albeit before we understood the extent of LGT that occurs between *Wolbachia* and its hosts. We now know that there are multiple copies of the *Wolbachia* genome integrated into the *D. ananassae* genome with insertional mutagenesis of the LGT by retrotransposons active in *D. ananassae* (Klasson *et al.* 2014; Dunning Hotopp *et al.* 2007), making it nearly impossible to resolve the sequence and organization with next generation sequencing techniques or bacterial artificial chromosomes. Therefore, we used fluorescence *in situ* hybridization and microscopy to demonstrate the likely location is the Muller F element (Klasson *et al.* 2014). The methods Leung *et al.* (2017) used to make the improvements to the contigs attributed to the Muller F element would not be sufficient to assemble the massive LGT from *Wolbachia* into *D. ananassae*, and, thus, are not sufficient to contradict this result.

This highlights the complexity of genome sequencing projects and their interpretation. While genomes are often presented and considered as final, static, and definitive objects, the experiments undertaken to obtain these sequences have nuances and/or assumptions that often need to be considered for proper interpretation of subsequent results. This also highlights that, as our knowledge about genome biology improves, we may need to reconsider and reanalyze earlier genomes removing the prejudice introduced from now defunct paradigms. As such, we recommend that the sequences deposited at the NCBI under PRJNA13365 should not be attributed to *Wolbachia* endosymbiont of *D. ananassae*, but should have their taxonomy reclassified by NCBI as “Unclassified sequences.”
